# Acute exacerbation of idiopathic pulmonary fibrosis disease: a diagnosis model in China

**DOI:** 10.1186/s40001-024-01791-x

**Published:** 2024-03-25

**Authors:** Liye Meng, Jun Xiao, Li Wang, Zhuochun Huang

**Affiliations:** 1 Department of Laboratory Medicine, West China Hospital, Sichuan University, Chengdu, 610041 China; 2 Department of Pulmonary and Critical Care Medicine, West China Hospital, Sichuan University, Chengdu, 610041 China

**Keywords:** Acute exacerbation of idiopathic pulmonary fibrosis/AE-IPF, Diagnosis model, Disease severity, Risk prediction

## Abstract

**Objective:**

To develop and validate a diagnosis model to inform risk stratified decisions for idiopathic pulmonary fibrosis patients experiencing acute exacerbations (AE-IPF).

**Methods:**

In this retrospective cohort study performed from 1 January 2016 to 31 December 2022, we used data from the West China Hospital of Sichuan University for model development and validation. Blood test results and the underlying diseases of patients were collected through the HIS system and LIS system. An algorithm for filtering candidate variables based on least absolute shrinkage and selection operator (LASSO) regression. Logistic regression was performed to develop the risk model. Multiple imputation handled missing predictor data. Model performance was assessed through calibration and diagnostic odds ratio.

**Results:**

311 and 133 participants were included in the development and validation cohorts, respectively. 3 candidate predictors (29 parameters) were included. A logistic regression analysis revealed that dyspnea, percentage of CD4^+^  T-lymphocytes, and percentage of monocytes are independent risk factors for AE-IPF. Nomographic model was constructed using these independent risk factors, and the C-index was 0.69. For internal validation, the C-index was 0.69, and that indicated good accuracy. Diagnostic odds ratio was 5.40. Meanwhile, in mild, moderate, and severe subgroups, AE positivity rates were 0.37, 0.47, and 0.81, respectively. The diagnostic model can classify patients with AE-IPF into different risk classes based on dyspnea, percentage of CD4^+^  T-lymphocytes, and percentage of monocytes.

**Conclusion:**

A diagnosis model was developed and validated that used information collected from HIS system and LIS system and may be used to risk stratify idiopathic pulmonary fibrosis patients experiencing acute exacerbations.

**Supplementary Information:**

The online version contains supplementary material available at 10.1186/s40001-024-01791-x.

## Introduction

Idiopathic pulmonary fibrosis (IPF) is the most common of the idiopathic interstitial pneumonias, characterized by severe pulmonary tissue fibrosis without a known pathogenesis. And the mean survival time after diagnosis is 2–3 years [1, 2]. It is a devastating condition that carries a prognosis worse than that of many cancers [2, 3]. Except for lung transplantation, there are very few treatment options for a patient with a fibrotic disease [4]. IPF patients frequently suffer an acute exacerbation (AE) of respiratory failure, also known as AE-IPF [5, 6]. It can cause severe acute hypoxemic respiratory failure, sharing common features with acute respiratory distress syndrome (ARDS).

In a retrospective study of 461 IPF cases, AE-IPF was the most frequent cause for the deterioration of IPF, accounting for 55.2% of the cases [7]. In another review, the ICU mortality of AE-IPF was from 56% to 100%, meanwhile the hospital mortality was 56% to 100% [5]. It is crucial to accurately identify the high risk of AE-IPF patients for optimizing their management and providing personalized care.

Currently, biological data grow and become more complex, machine learning is being applied to build predictive and informative models of these processes [8]. For example, analyzing primary care data to develop and validate a prognostic model for assessing methotrexate toxicity in immune-mediated inflammatory diseases [9]. According to percentage predicted forced vital capacity and PaO_2_/FiO_2_ levels, an interstitial lung disease prognosis model was developed for patients with AE rheumatoid arthritis [10]. The gender, age, physiology (GAP) model was used to determine disease severity in the idiopathic pulmonary fibrosis [11]. Notably, blood test results are the easiest to obtain.

There have been reports of immune cell disorders in patients with IPF [12, 13]. It has been demonstrated that certain patients with similar pulmonary fibrosis, such as COVID-19, have immune cell infiltration in the lungs and a decrease in CD4^+^ T and CD8^+^ T in the peripheral blood [14]. In this study, immunological data from the electronic medical record system of West China Hospital of Sichuan University were used to develop and validate a diagnosis model for AE-IPF. For patients with AE-IPF, it would be beneficial to predict the likelihood of clinically significant abnormal blood test results. In order to better understand this risk, we developed and validated a diagnosis model to estimate the risk of clinically significant idiopathic pulmonary fibrosis patients experiencing acute exacerbations.

## Methods

In this retrospective cohort study performed from 1 January 2016 to 31 December 2022, we used data from the electronic medical record system of West China Hospital of Sichuan University (WCHSCU) for model development and validation. As WCHSCU is the largest general hospital in Southwest China and is representative of the patients for the database. The electronic medical record system includes information on demographic factors, basic diseases (hypertension, diabetes, cardiovascular disease, etc.), complications at the onset (pulmonary hypertension, gastroesophageal reflux disease, etc.), blood test results, and smoking status. According to TRIPOD guidelines, this study is transparently reporting multivariable prediction models for individual diagnosis [15].

## Study population

This retrospective study enrolled consecutive IPF patients who were hospitalized in the West China Hospital of Sichuan University between 1 January 2016 and 31 December 2022. 444 patients were selected to construct the prediction model. 45.5% of patients received anti-fibrotic therapy, meanwhile, 56.3% of patients received oxygen supply (Additional file 1: Table S1). Data should be collected on AE-IPF diagnosis if it existed; otherwise, data should be collected on the first exam the patient underwent.

IPF was defined by the presence of bilateral reticular opacities with/without traction bronchiectasis on chest high-resolution computed tomography (HRCT). The diagnosis of IPF was confirmed through clinical features, imaging data, and medical history according to the diagnostic criteria for IPF [16]. AE-IPF was diagnosed according to the 2016 AE-IPF International Working Group report [17]. The diagnostic criteria for AE-IPF were as follows: (1) the past or presence of fibrosing ILD on HRCT; (2) acute worsening or development of deteriorative acute dyspnea typically within 1 month; (3) A new bilateral ground-glass opacity and/or consolidation superimposed on fibrosing ILD on HRCT; and (4) Unaccounted deterioration unrelated to cardiac failure or excessive fluid accumulation. This study was approved by the Clinical Trials and Biomedical Ethics Committee of West China Hospital of Sichuan University (approval number: No. 366 in 2022).

### Variable collection

Variable selection would be based on physicians' opinions and immune disorders of the disease in subsequent analyses. Data pertaining to the following variables were collected from the medical records: clinical data, including the age, hypertension, etc.; laboratory data at AE onset, including 29 indicators (Table 1) and outcomes.

**Table 1:** Summary characteristics of final study cohort overall and separated into distinct sub-cohorts for internal–external cross-validation

Variables and categories	Overall study cohort (*n* = 444)	Development cohort (*n* = 311)	validation cohort (*n* = 133)
AE-IPF, *n* (%)	247(55.6)	171(55.0)	76(57.1)
Age at onset (years)	67.9 ± 9.8	67.6 ± 9.5	68.8 ± 7.1
Gender (male,%)	357(80.4)	248(79.7)	109(82.0)
Dyspnea, *n* (%)	119(26.8)	84(27.0)	35(34.01)
Comorbidities:			
Infection of lungs, *n* (%)	291(65.5)	207(66.6)	84(63.2)
Renal dysfunction, *n* (%)	19(4.3)	12(4.0)	7(5.3)
Hypertension, *n* (%)	119(26.8)	86(27.7)	33(24.8)
Diabetes, *n* (%)	104(23.4)	68(21.9)	36(27.1)
Admission indicators:			
Temperature, ℃	36.5(36.3–36.7)	36.5(36.3–36.7)	36.5(36.3–36.6)
Breaths, minute	20.0(20.0–22.0)	20.0(20.0–22.0)	20.0(20.0–22.0)
Pulse, minute	87.0(80.0–98.0)	87.0(80.0–98.0)	89.0(78.5–98.0)
Systolic pressure, mmHg	126.5(113.0–138.0)	126.0(113.0–138.0)	128.0(116.5–138.0)
Diastolic pressure, mmHg	77.0(69.00–85.00)	76.0(69.0–85.0)	78.0(71.5–85.0)
Immune status at admission			
Leucocytes, 10^9^/L	7.3(6.0–9.2)	7.4(6.11–9.3)	7.1(5.8–8.7)
Neutrophil, %	66.1(57.50–76.50)	66.0(57.9–76.5)	66.5(56.7–76.6)
Lymphocyte, %	22.1(15.3–29.9)	22.2(15.3–29.4)	21.2(15.3–31.1)
Monocyte, %	6.9(5.0–8.6)	6.9(5.3–8.6)	6.6 ± 3.0
Neutrophil, 10^9^/L	4.8(3.6–6.6)	4.9(3.7–6.5)	4.6(3.4–6.7)
Lymphocyte, 10^9^/L	1.6(1.1–2.0)	1.7 ± 0.8	1.6(1.2–2.0)
Monocyte, 10^9^/L	0.5(0.3–0.7)	0.5(0.3–0.7)	0.5(0.3–0.7)
CD3^+^ T-lymphocyte, %	67.1(57.4–75.5)	66.9(56.3–75.4)	68.3(59.4–75.8)
CD4^+^ T-lymphocyte, %	34.6(26.7–42.2)	34.2 ± 10.9	34.0 ± 11.4
CD8^+^ T-lymphocyte, %	26.4(20.0–34.8)	26.4(19.2–33.8)	26.3(21.4–37.9)
CD4/CD8	1.3(0.8–1.9)	1.3(0.9–2.0)	1.2(0.8–1.8)
Immunoglobulin G, g/L	13.9(11.7–17.0)	13.9(11.6–17.1)	13.9(12.1–16.8)
Immunoglobulin A, g/L	2.9(2.2–3.8)	2.9(2.2–3.7)	3.0(2.1–4.1)
Immunoglobulin M, g/L	1.0(0.7–1.4)	1.0(0.7–1.4)	0.9(0.7–1.4)
Total protein, g/L	68.5 ± 6.5	68.3 ± 6.3	68.9 ± 7.1
Albumin, g/L	38.7(35.2–41.7)	38.6(34.9–41.7)	38.7 ± 4.8
CO_2_	23.5(21.9–26.0)	23.5(22.1–26.0)	23.5(21.5–26.0)

### Final predictor selection

We employed a statistical consolidation technique called the Least Absolute Shrinkage and Selection Operator (LASSO) to combine all variables. The core principle of LASSO involved applying a penalty function to shrink the regression coefficient of each variable within a specific range, irrespective of its statistical significance. Variables with a coefficient of 0 were eliminated, resulting in a final set of optimal and representative variables. As a result, the coefficients were optimized, and less significant variables were excluded [18].

### Sample size

For model development, we adopted Riley and colleagues’ formulae to construction of the prediction model [19]. Using above formulae, we determined that to minimize model overfitting (parameters: a shrinkage factor is 0.1, an anticipated *R*^2^_cs_ is 0.3), and we required a minimum sample size of 103 participants based on a maximum of 29 parameters. The variables tested were adjusted for an additional 29 covariate(s), which had a combined *R*^2^ of 0.3 by themselves.

### Statistical analysis

SPSS 20.0 (SPSS Inc., Chicago, IL, USA) and R Statistical Software (v4.1.2, R Foundation for Statistical Computing, Vienna, Austria) were used for data analysis. Diagnostic odds ratio (DOR), odds ratio of positive labeling for condition positive versus negative, was calculated to predict the degree of discrimination of the prediction model [20]. In order to reflect the AE-IPF risk stratification effect, the values at risk were divided into mild, moderate, and severe subgroups, and the frequency of AE was calculated separately. Finally, an internal validation method was used to test the stability of the prediction model. The “rms” package was used to calculate the C-index in final model. Multiple imputation handled missing predictor data on IgG, IgA, IgM and CO_2_ using chained equations [9]. The “Caret” package was used to randomly assign 444 patients to development cohort and validation cohort in a 7:3 ratio.

#### Construction of the prediction model

Least absolute shrinkage and selection operator (LASSO) regression was used to filter candidate variables for development of prediction model. All 3 candidate variables (29 parameters) were included in the final model. Rubin's rule was applied to the imputed datasets to estimate the coefficients of each predictor. The development data were used to formulate the risk equation for predicting an individual's risk of AE-IPF. The risk of AE-IPF was estimated along with the estimated regression coefficients (*β*) and the individual’s variables (*X*) at onset, and predicted the risk = exp(*βX*)/(1 + exp(*βX*)), where *βX* is the linear predictor, equaled to *β*_1_*X*_1_ + *β*_2_*X*_2_ + *β*_3_*X*_3_.

Predictive model was constructed using multivariate logistic regression based on demographic variables, underlying disease, co-morbidities and laboratory blood tests together. 3 candidate predictors (29 parameters) were included in the logistic model, and the coefficients of each predictor were estimated and combined using Rubin’s rule across the imputed datasets. The stability of the models was verified by tenfold cross-validation.

### Model internal validation

The calibration curve that assessed the agreement between predicted and observed risks across the entire range. An ideal calibration curve would closely follow the 45° line, indicating perfect agreement between predicted and observed outcomes.

Internal validation was performed using bootstrapping with replacement on 116 samples of the valid data to correct for overfitting. The full model was fitted to each bootstrap sample, and its performance was measured on both the bootstrap samples (apparent performance) and the original samples (test model performance). Model optimism, which reflects overestimation due to overfitting, was calculated as the difference between the test performance and apparent performance.

The optimism-adjusted estimates of the model's performance for the original model were calculated by subtracting the optimism from the original apparent performance. The graph provided insights into the model's calibration performance and accounted for overfitting to obtain more accurate estimates of model performance.

## Results

### Patients and clinical characteristics

A total of 444 patients were retrospected in this study, among which 311 patients were enrolled in the candidate variable selection cohort (development cohort), and 133 patients were enrolled in the internal validation cohort. There was statistically significant difference in the frequency of dyspnea between the development cohort (17.77%) and validation cohort (34.01%), but the proportion of dyspnea in AE-IPF patients was no statistically significant difference.

### Development of prediction model

**Fig. 1:**
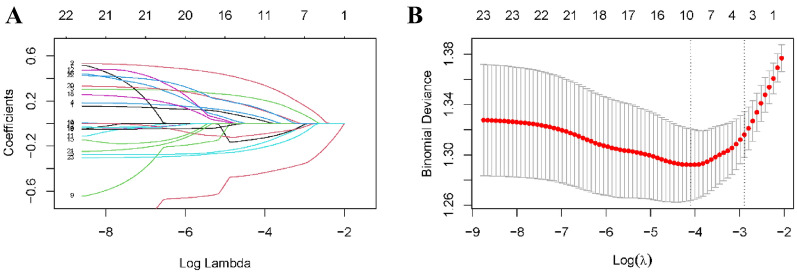
Filtering candidate variables by LASSO. **A** Shows the Lasso variable coefficient plot. Each curve in the figure represents the trajectory of a variable's coefficient. The vertical axis represents the values of the variable coefficients. The bottom horizontal axis, *λ*, is the parameter controlling the severity of the penalty, and the top horizontal axis represents the number of non-zero coefficients in the model under the penalty parameter. **B** Shows the parameter adjustments in LASSO. Fine-tuning the LASSO model's regularization parameters involves screening *λ* using a tenfold cross-validation approach. A dashed vertical line is positioned at 1 standard error (1-SE standard) from the minimum and maximum values of *λ*. *λ*.1se represents the parameter value associated with a model that delivers optimal performance while utilizing the fewest number of variables

**Fig. 2:**
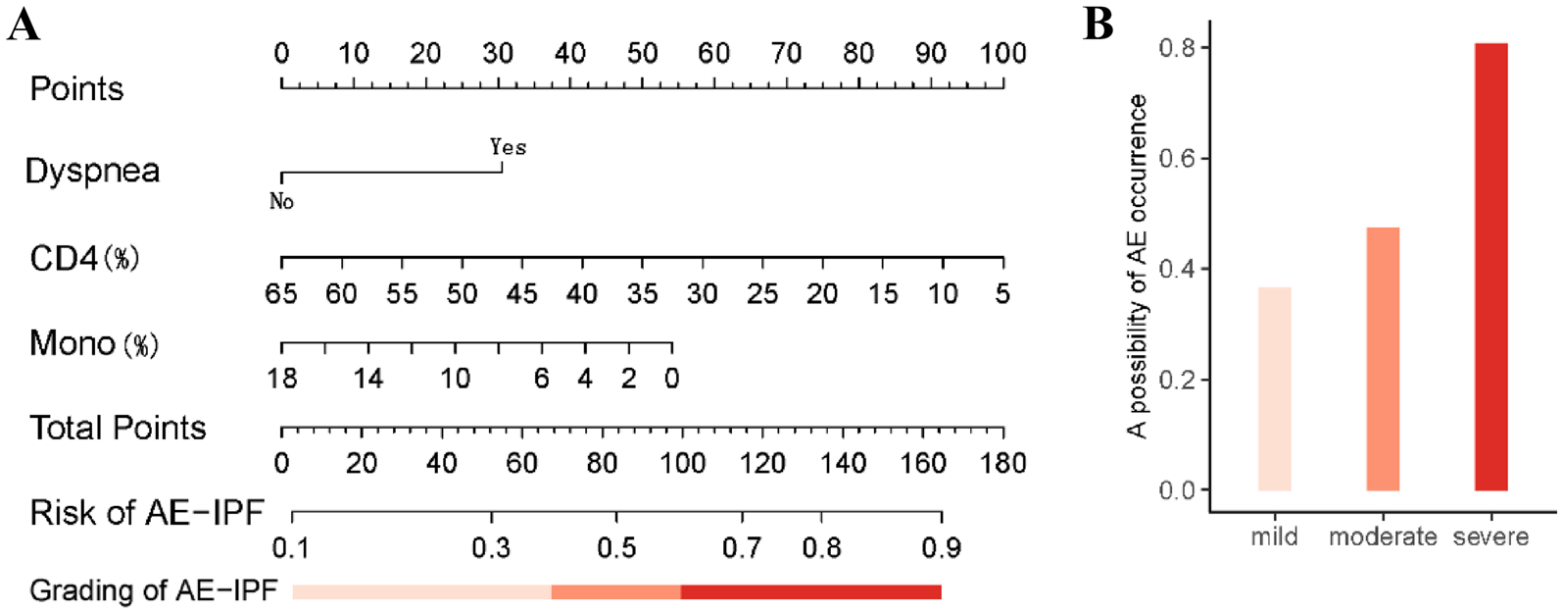
**A** Shows nomogram to predict the incidence of AE-IPF. **B** Shows the occurrence of AE-IPF within differ grading subgroups. CD4 (%): percentage of CD4 ^+^ T-lymphocytes. Mono (%): percentage of monocytes

Based on the results of the LASSO regression, we determined three efficiency predictive factors for AE-IPF (Fig. 1). A nomographic risk prediction model was meticulously developed to enable precise estimation of AE-IPF risk based on three distinct variables (Fig. 2A). Scales were used to score each variable, with total score ranging from 0 to 180. A higher score on the AE-IPF risk axis indicated a greater chance that a patient with IPF will develop AE-IPF (Fig. 2B). Dyspnea, percentage of CD4 ^+^ T-lymphocytes, and percentage of monocytes at admission were strong predictors of AE-IPF, with hazard ratios of 2.28 (95% confidence interval 1.43 to 3.70), 0.96 (0.94 to 0.97), and 0.92 (0.86 to 0.99), respectively (Table 2).

**Table 2:** Model parameter

Variables	β coefficient	Std. error	Hazard ratio (95%CI)
dyspnea	0.82612	0.24115	2.28 (1.43, 3.70)
CD4 ^+^ T-lymphocytes(%)	-0.04518	0.01023	0.96(0.94, 0.97)
monocytes(%)	-0.08150	0.03713	0.92(0.86, 0.99)

Risk score = exp(βX)/(1 + exp(βX)), βX = 0.82612 × dyspnea -0.04518 × CD4 ^+^ T-lymphocytes(%) -0.0815 × monocytes(%) + 2.13781

### Model performance in validation cohort

By plotting the agreement between predicted and observed risks (the calibration of the model) across the entire range, a smoothed non-parametric calibration curve was used to assess the model's calibration performance. Ideally, the curve should be close to the 45° line (calibration slope of 1).

**Fig. 3:**
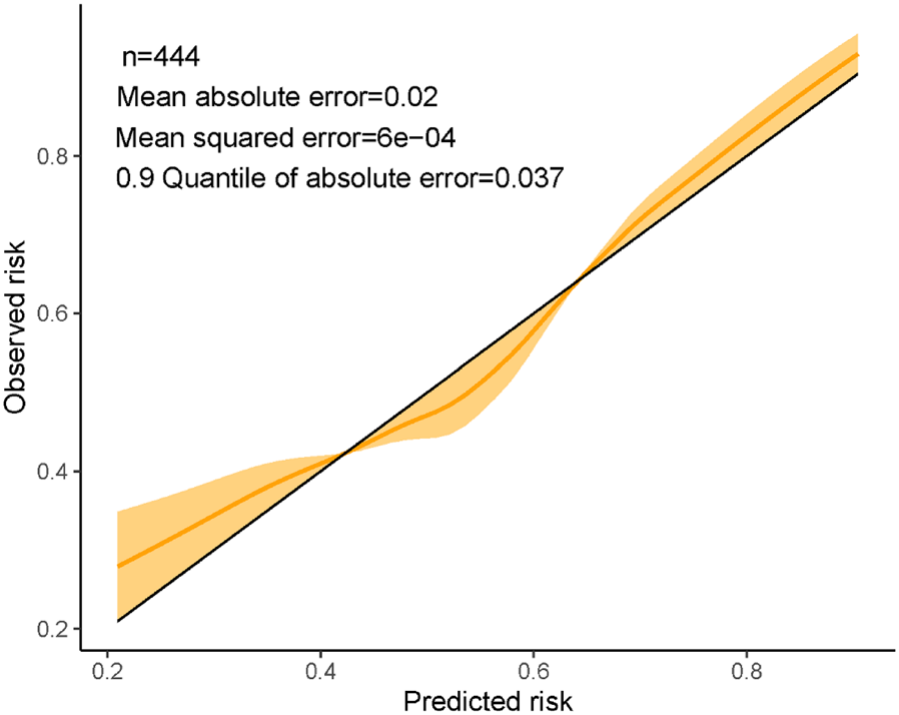
Calibration of a diagnosis model for AE-IPF. Black line reflected ideal prediction, while orange line showed actual prediction

The calibration slope was 0.99 (95% confidence interval 0.98 to 0.99). The calibration plot showed reasonable correspondence between observed and predicted risk (Fig. 3). Diagnostic odds ratio, a valuable measure because it takes into account both sensitivity and specificity of a diagnostic test, was used to assess the accuracy of this model. Diagnostic odds ratio of this model was 5.40, indicating a good diagnostic performance.

## Discussion

Although the cause of AE-IPF is not very clear, early identification and treatment interventions might significantly improve the patient's condition. Moreover, AE is a common cause of death in IPF patients, and has a significant impact on the short and long-term outcomes of IPF patients [21, 22]. Therefore, a prediction of AE-IPF is crucial. Unfortunately, there is no reliable model for predicting AE-IPF. The present study created a model to predict the risk of AE-IPF and provided a reference for clinical prediction.

Our findings demonstrated that dyspnea, percentage of CD4 ^+^ T-lymphocytes, and percentage of monocytes can be independently important risk factors for AE among patients with IPF. It can be used to stratify patients with acute exacerbations of idiopathic pulmonary fibrosis. Our model used predictors that are more common, accessible, and convenient for primary health care and community hospitals. Thus, monitoring AE risk was convenient for IPF during follow-up.

While the predictive factors employed in the model were not exclusive to AE-IPF and do not serve as definitive diagnostic criteria for AE-IPF, nonetheless, they could provide insights into the respiratory function and in vivo immune status of patients with AE-IPF. The presence of dyspnea is a common deteriorating factor in patients with IPF and is an important basis for the diagnosis of AE-IPF [17]. In a retrospective, multicenter study, increased CD14 ^+^ classical monocyte percentages were found to be significantly associated with survival in short-term, lung transplant-free IPF patients [3]. Kawamura et al. reported that monocytes count were found to be a risk factor for AE in patients with IPF [23]. However, the study focused more on pulmonary CT manifestations and changes in pulmonary ventilation function in patients with IPF, as well as the relatively small number of investigators included. Our study placed a greater emphasis on monitoring peripheral immune function changes during acute exacerbations in IPF patients. It aimed to investigate the immune-related risk factors associated with AE. Accordingly, our study can serve as a complementary research.

A number of studies had shown that patients with IPF had an increase in CD4 ^+^ and CD8 ^+ ^T cells in the lungs and in their BAL [13, 24]. Decreased CD4^+^ CD28^+^ T cells, decreased Treg cells had been revealed with worse prognosis and more severe disease in IPF patients by several studies [25, 26]. In our scoring model, a significant portion of CD4^+^ T cells featured prominently, indicating a potential strong association between CD4^+^ T cell percentages and AE. We hypothesized that the reduction in peripheral blood CD4^+^ T cells might be attributed to their recruitment to the lungs, where they potentially engaged in the pulmonary inflammatory response.

Misharin and colleagues validated the involvement of monocytes in IPF using a mouse mode. Lung injury triggered the recruitment of monocytes into the lung, where they underwent differentiation into monocyte-derived alveolar macrophages (Mo-AMs). These Mo-AMs played a crucial role in driving fibrosis development following intratracheal administration of bleomycin or an adenoviral vector encoding active TGF-β [27, 28]. Our study further confirmed that the occurrence of acute exacerbations in IPF leads to significant alterations in the immune status. Consequently, the current model can provide valuable information about AE-IPF cases.

### Strengths of this study

Strengths of this study included the use of real-world data from the largest general hospital in Southwest China. Consequently, the result exhibited extensive potential for general applicability. Regarding variable selection, factors deemed risky by the physician and documented in literature were included. At the same time, the risk factors were available in electronical healthy records. Therefore, the results possessed potential value for clinical application.

### Limitations of this study

Certain constraints of this study warranted contemplation. Lung function ventilation tests were added without incorporating, such as forced vital capacity (FVC)and total lung capacity (TLC) [29], for only a small subset of patients having these records in the electronic medical records. Some patients had received treatment before being transferred to our medical institutions, and there was no pulmonary function records in our electronic medical records. For certain patients with severe conditions, undergoing pulmonary function tests was not advisable. Therefore, it would be unreasonable to use imputation methods for subsequent analysis.

## Conclusion

A diagnosis model that can be used in clinical practice for predicting the likelihood of AE in patients with IPF had been developed and internally validated. It was recommended that additional validation studies be conducted with populations outside of China and that fewer predictor parameters be taken into account.

### Supplementary Information

**Additional file 1: Table S1.** Summary therapy information of final study cohort.

## Data Availability

The raw datasets used in this study are available from the corresponding author on reasonable request.
