# Supplementary material for: Acute exacerbation of idiopathic pulmonary fibrosis disease: a diagnosis model in China
Source: Eur J Med Res. 2024 Mar 25;29:198. doi: 10.1186/s40001-024-01791-x (PMC10962171; doi:10.1186/s40001-024-01791-x)
Supplement: Supplementary file 1 — Additional file 1: Table S1. Summary therapy information of final study cohort. [file 40001_2024_1791_MOESM1_ESM.docx]

**Table S1 Summary therapy information of final study cohort.**

| variables and categories | Overall study cohort  (n=444) | Development cohort  (n=311) | validation cohort  (n=133) |
| --- | --- | --- | --- |
| Therapy: |  |  |  |
| N-acetylcysteine, n (%) | 70(15.8) | 49(15.6) | 21(15.8) |
| Pirfenidone, n (%) | 108(24.3) | 73(24.8) | 35(26.3) |
| Nintedanib, n (%) | 24(5.4) | 17(5.5) | 7(5.3) |
| Oxygen: |  |  |  |
| noninvasive ventilation, n (%) | 25(5.6) | 17(5.5) | 8(6.0) |
| mask, n (%) | 10(2.3) | 6(2.0) | 4(3.0) |
| Others | 215(48.4) | 153(49.2) | 62(46.6) |
